# A Two-Stage Mutual Information Based Bayesian Lasso Algorithm for Multi-Locus Genome-Wide Association Studies

**DOI:** 10.3390/e22030329

**Published:** 2020-03-13

**Authors:** Hongping Guo, Zuguo Yu, Jiyuan An, Guosheng Han, Yuanlin Ma, Runbin Tang

**Affiliations:** 1 Key Laboratory of Intelligent Computing and Information Processing of Ministry of Education and Hunan Key Laboratory for Computation and Simulation in Science and Engineering, Xiangtan University, Xiangtan 411105, China; guohongping0501@163.com (H.G.); hangs@xtu.edu.cn (G.H.); 201590110068@smail.xtu.edu.cn (Y.M.); 201831510085@smail.xtu.edu.cn (R.T.); 2 School of Mathematics and Computer Science, Hanjiang Normal University, Shiyan 442000, China; 3 School of Electrical Engineering and Computer Science, Queensland University of Technology, Brisbane, QLD 4001, Australia; 4 Centre for Tropical Crops and Biocommodities, Queensland University of Technology, Brisbane, QLD 4001, Australia; j.an@qut.edu.au

**Keywords:** GWAS, Pearson correlation, mutual information, feature screening, Bayesian Lasso

## Abstract

Genome-wide association study (GWAS) has turned out to be an essential technology for exploring the genetic mechanism of complex traits. To reduce the complexity of computation, it is well accepted to remove unrelated single nucleotide polymorphisms (SNPs) before GWAS, e.g., by using iterative sure independence screening expectation-maximization Bayesian Lasso (ISIS EM-BLASSO) method. In this work, a modified version of ISIS EM-BLASSO is proposed, which reduces the number of SNPs by a screening methodology based on Pearson correlation and mutual information, then estimates the effects via EM-Bayesian Lasso (EM-BLASSO), and finally detects the true quantitative trait nucleotides (QTNs) through likelihood ratio test. We call our method a two-stage mutual information based Bayesian Lasso (MBLASSO). Under three simulation scenarios, MBLASSO improves the statistical power and retains the higher effect estimation accuracy when comparing with three other algorithms. Moreover, MBLASSO performs best on model fitting, the accuracy of detected associations is the highest, and 21 genes can only be detected by MBLASSO in *Arabidopsis thaliana* datasets.

## 1. Introduction

Genome-wide association study (GWAS) has evolved to be an essential technology for exploring the genetic mechanism of complex traits [1]. It concentrates on identifying the significant single nucleotide polymorphisms (SNPs) associated with the given traits. In past years, several single-locus GWAS methods have been developed [1,2,3,4,5], and have detected a few variants among various traits successfully. However, they still have some drawbacks, such as the combined effects of multiple loci are ignored and the threshold in multiple test correction is hard to be determined [6].

To address these drawbacks, some classical high-dimensional statistical methods were well used in GWAS when the number of SNPs is not far more than that of individuals, such as the least absolute shrinkage and selector operator (Lasso) [7], the elastic net [8], and Bayesian Lasso [9,10]. However, the current situation is the opposite, because the number of SNPs is much larger than that of individuals. In the case of ultrahigh-dimensional data, the aforementioned methods will fail due to the internal computational complexity. Fortunately, Fan and Lv [11] proposed a two-stage feature screening (or variable selection) method. The main idea of this method is: The dimension of features are firstly cut down by sure independence screening (SIS), and then a certain popular high-dimensional feature screening method (such as Lasso, the smoothly clipped absolute deviation (SCAD) [12], or the adaptive Lasso [13]) is used to select significant features and estimate regression coefficients simultaneously. The extension of SIS is iterative sure independence screening (ISIS), which can revive those non-negligible features that are single uncorrelated while indirectly correlated to the respond variables [11]. Instead of the Pearson correlation based SIS, statisticians have exploited some other SIS methods from different measurements, such as rank correlation [14], the distance correlation [15], the partial correlation [16] and so on. Among these methods, Pearson correlation and distance correlation based screening have been applied in GWAS successfully [6,17], and some genes associated with crop quantitative traits such as rice salt-tolerance and poplar growth have been identified [18,19]. ISIS expectation-maximization Bayesian LASSO (ISIS EM-BLASSO) [6] selects potentially associated SNPs in single-objective screening methodology based on the Pearson correlation between the SNPs and phenotype. In reality, the intrinsic heterogeneity is likely to be present in big data [20], thus the characterization of correlations via multi-objective method can bring higher power [21]. Although two-side high-dimensional genome-wide association studies (2HiGWAS) [17] efficiently selects the associated SNPs by combining Pearson correlation and distance correlation, the computational burden of constructing distance correlation is very high.

Since mutual information can detect broader classes of relationships [22], and the computational complexity is relatively low [23]. We propose to modify the screening method in the first stage of ISIS EM-BLASSO to a multi-objective one, which is based on the combination of Pearson correlation and mutual information. Then EM-Bayesian Lasso (EM-BLASSO) [10] is applied to further select SNPs and estimate the effects by shrinking the weak effects to zero, and likelihood ratio test is used to identify the true quantitative trait nucleotides (QTNs), these procedures are the same as those in the second stage of ISIS EM-BLASSO (also denoteds EM-BLASSO). We call our method a two-stage mutual information based Bayesian Lasso (MBLASSO). In order to validate the effectiveness of our method, we compare it with three GWAS methods, EM-BLASSO [10], ISIS EM-BLASSO [6] and genome-wide efficient mixed model association (GEMMA) [5]. EM-BLASSO represents the single-stage GWAS method without pre-screening, ISIS EM-BLASSO is a typical two-stage GWAS method using only Pearson correlation screening, and GEMMA is a golden standard GWAS method widely used for comparison.

## 2. Materials and Methods

### 2.1. Statistical Framework

In this study, we consider the linear mixed genetic model [6] as follows:

(1) y=1μ+Qα+Xβ+ε

where y is a n×1 phenotypic vector of quantitative trait, and *n* is the number of individuals; 1 is a n×1 vector in which every element is equal to 1, and μ is the overall mean; $Q=(Q_{1},Q_{2},\ldots ,Q_{q})$ is a n×q matrix of fixed effects, such as the population structure, and *q* is the number of fix effects; α is a q×1 vector of fixed effects; and *X* is a n×p matrix of SNP genotype values. For each SNP, homozygous genotype are coded as 1, and −1, respectively, and the heterozygous ones are indicated by 0. *p* is the number of presumed QTNs, β is the QTN effects, and $\varepsilon ∼MVN_{n}\left(0,\sigma_{e}^{2}I\right)$ is a n×1 vector of residual error.

### 2.2. Simulation Experiments

To assess the performance of methods, we considered simulation scenarios based on the *Arabidopsis thaliana* datasets consisting of 216,130 SNPs, 199 accessions, and 107 phenotype traits [24]. For genotype simulation, we randomly selected 10,000 SNPs, 2000 for each of the five chromosomes, i.e., 11,226,256–12,038,776 bp on Chr.1, 5,045,828–6,6412,875 bp on Chr.2, 1,916,588–3,196,442 bp on Chr.3, 2,232,796–3,3143,893 bp on Chr.4, and 19,999,868–21,039,406 bp on Chr.5. Additionally, we generated the phenotype simulation data with sample size 199 from three different scenarios, and undertook 1000 times for each simulation. Six QTNs were assumed to be genuine; their heritabilities were set as 0.10, 0.05, 0.05, 0.15, 0.05, and 0.05, respectively; and their allelic frequencies we are all nearly 0.30. Both the overall mean and residual variance we are set as 10.0, and the positions and effects of the six QTNs are shown in Tables S1–S3. The genotype and phenotype simulations were the same as those used by Wang et al. [25].

The first model (only six QTNs’ additive effects) is: $y=\mu +\sum_{i=1}^{6}x_{i}b_{i}+\varepsilon$, $\varepsilon ∼MVN_{n}\left(0,\sigma_{e}^{2}I\right)$. The second model (six QTNs’ additive effects plus polygenic effect) is: $y=\mu +\sum_{i=1}^{6}x_{i}b_{i}+u+\varepsilon$, $u∼MVN_{n}\left(0,\sigma_{pg}^{2}K\right)$, $\varepsilon ∼MVN_{n}\left(0,\sigma_{e}^{2}I\right)$, and K is the kinship matrix. Set $\sigma_{pg}^{2}=2$, thus $h_{pg}^{2}=0.092$. The third model (six QTNs’ additive effects plus three other pairs of QTNs’ epistatic effects) is: $y=\mu +\sum_{i=1}^{6}x_{i}b_{i}+\sum_{j=1}^{3}\left(A_{j}\#B_{j}\right)b_{jj}+\varepsilon$, $\varepsilon ∼MVN_{n}\left(0,\sigma_{e}^{2}I\right)$, # denotes Hadamard product (element-wise multiplication), three other pairs of epistatic QTNs (unrelated to the six true QTNs) are placed on 3063784bp (Chr.4) and 5227063bp (Chr.2), 5986135bp (Chr.2) and 2031781bp (Chr.3), and 2668059bp (Chr.3) and 11824678bp (Chr.1), respectively. Each pair of QTNs was set with $\sigma_{epi}^{2}=1.25$, thus $h_{epi}^{2}=0.05$.

### 2.3. Real Data and Preprocessing

We used four flowering-time related traits of *Arabidopsis thalina* datasets [24,26] for analysis. The four traits are days to flowering time under long days with vernalization (LDV), days to flowering time under short days with vernalization (SDV), days to flowering time under long days with two weeks vernalization (2W), and days to flowering time under long days with four weeks vernalization (4W), respectively. We removed the SNPs with minor allele frequency (MAF) less than 0.01, and 178376 SNPs remained ultimately. For phenotypes, we deleted the individuals with missing phenotype value, thus 168, 159, 152 and 119 individuals were reserved for each of the four traits LDV, SDV, 2W and 4W, respectively, and then a logarithmic transformation was performed to each phenotype value. Due to the strong population structure in *Arabidopsis thaliana*, we were obliged to eliminate the impact of population structure. We reorganized the SNP genotype data via the software PLINK (Version 1.09) [27] at first, then chose a suitable value for population number *q* from 1 to 5 with the minimum cross-validation error, and calculated the population structure matrix *Q* synchronously by using the software ADMIXTURE (Version 1.3) [28], and finally corrected the primary phenotype vector y by $Q_{j},j=1,2,\ldots ,q$, whose effects $\hat{\alpha_{j}}$ were estimated by least-square method. Therefore, the corrected phenotype vector is:

(2) $$y^{{}^{\prime }}=y-\sum_{j=1}^{q}Q_{j}\hat{\alpha_{j}}=1\mu +\sum_{i=1}^{p}X_{i}\beta_{i}+\varepsilon$$

### 2.4. Mutual Information

Mutual information proposed by Shannon [29] is based on the concept of entropy and has been widely used in feature selection [23]. Given two discrete random variables *X* and *Y*, the mutual information of *X* and *Y* is defined as:

(3) I(X;Y)=H(X)+H(Y)−H(X,Y)

where H(X) is the entropy of *X* and H(X,Y) is the joint entropy of *X* and *Y*. They can be specified as:

(4) $$H\left(X\right)=-\sum_{x}p\left(x\right)\cdot \log p(x)$$

(5) $$H\left(X,Y\right)=-\sum_{x,y}p(x,y)\cdot \log p(x,y)$$

where p(x)=P(X=x) is the marginal probability density function, and p(x,y)=P(X=x,Y=y) is the joint probability density function. Mutual information can also be defined as:

(6) $$I\left(X,Y\right)=H\left(X\right)-H\left(X|Y\right)=H\left(Y\right)-H\left(Y|X\right)=\sum_{x,y}p\left(x,y\right)\cdot \log\frac{p(x,y)}{p(x)p(y)}$$

where H(X|Y) is the conditional entropy of *X* given *Y*. We calculated the mutual information by using the matlab package “MutualInfo” (Version 0.9) written by Peng et al. [23].

In fact, mutual information can be illustrated as the amount of information one random variable contained in another random variable. The larger the mutual information is, the stronger correlation between the two random variables is. In GWAS, we consider the phenotypic vector as one random variable, and the genotype vector of a SNP as another random variable. In this way, we can calculate the mutual information between each of the SNPs and phenotype.

### 2.5. SCAD

SCAD is a penalized likelihood approach that enables to selecting variables and estimating coefficients simultaneously due to its Oracle properties [12]. The objective function ξ is:

(7) $$\xi_{\lambda ,\gamma }\left(\beta \right)=argmin_{\beta }\left(\sum_{i=1}^{n}{\left(y_{i}-\sum_{j=1}^{p}\left(X_{ij}\beta_{j}\right)\right)}^{2}+\sum_{j=1}^{p}\rho_{\lambda ,\gamma }\left(\right|\beta_{j}\left|\right)\right)$$

where $\beta ={\left(\beta_{1},\beta_{2},\ldots ,\beta_{p}\right)}^{T}$ is the regression coefficient vector to be estimated and, λ and γ are penalty and shrinkage parameter, respectively, both of them are greater than 0. The former term of Equation (7) is the loss function, and the latter term is the penalty function defined by:

(8) $$\rho_{\lambda ,\gamma }\left(\beta_{j}\right)=\left\{\begin{array}{ccc} \lambda |\beta_{j}|, & \mathrm{if} & |\beta_{j}|<\lambda ,\\ \frac{-(|\beta_{j}{|}^{2}-2\gamma \lambda |\beta_{j}|+\lambda^{2})}{2(\gamma -1)}, & \mathrm{if} & \lambda \leq |\beta_{j}|<\gamma \lambda \;\mathrm{and}\;\gamma >2,\\ \frac{\left(\gamma +1\right)\lambda^{2}}{2}, & \mathrm{if} & \left|\beta_{j}\right|\geq \gamma \lambda . \end{array}\right.$$

γ=3.7 as suggested in the original study [12]. We performed SCAD by using the R package “ncvreg” from https://CRAN.R-project.org/package=ncvreg.

### 2.6. Likelihood Ratio Test

Likelihood ratio test is to compare the maximum of likelihood function in null hypothesis $H_{0}$ and alternative hypothesis $H_{1}$, and further determine whether the hypothesis is effective. LOD (log of odds) score is a statistic criterion used in likelihood ratio test. The definition is:

(9) $$LOD=log_{10}\left(\frac{l_{0}}{l_{1}}\right)=\frac{-2(L_{0}-L_{1})}{4.6052}$$

$l_{0}=e^{L_{0}}$, $l_{1}=e^{L_{1}}$, $L_{0}=L\left(\theta_{-k}\right)$ and $L_{1}=L\left(\theta \right)$ are the natural logarithms of the likelihood functions for null hypothesis $H_{0}:\beta_{k}=0$ and alternative hypothesis $H_{1}$, respectively, $\theta_{-k}=\left\{\beta_{1},\ldots ,\beta_{k-1},\beta_{k+1},\ldots ,\beta_{o}\right\}$ and $\theta =\{\beta_{1},\ldots ,\beta_{o}\}$, and *o* is the number of markers potentially associated with the trait. LOD≥3 was proposed to be the significant criterion in multi-locus GWAS [25], which is slightly stringent and equivalent to $P=Pr(\chi_{1}^{2}>3\times 4.6052)\approx 0.0002$. Under $H_{0}$, LOD×4.6052 follows a $\chi^{2}$ distribution with one degree of freedom. We set the significant criterion of MBLASSO, ISIS EM-BLASSO, and EM-BLASSO as LOD≥3, which is Bonferroni correction for GEMMA by referring the published study [30].

### 2.7. A Two-Stage Mutual Information Based Bayesian Lasso (MBLASSO) Method

**Figure 1:**
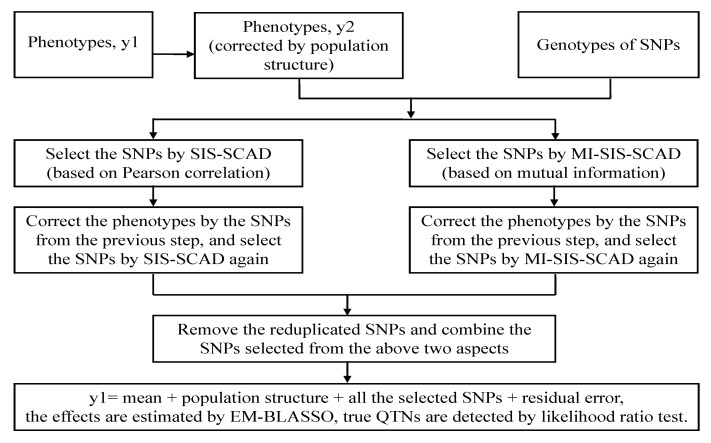
A flow chart of MBLASSO method.

On the whole, this procedure is a two-stage strategy for multi-locus GWAS. In the first phase, we used a modified ISIS approach based on Pearson correlation and mutual information to obtain a subset of SNPs, the elements of which can be divided into two types, separately. As to Pearson correlation screening, Type I includes those SNPs with strong correlated to phenotype, and Type II consists of those SNPs weak correlated while indirectly correlated to phenotype with some SNPs from Type I. For mutual information screening, Types I and II are similar as those in Pearson correlation screening. The first phase of our method can be considered to select SNPs from two different measurements. In the second phase, we adopted EM-BLASSO [10] to estimate the effects and select the SNPs with nonzero effect ($\geq 10^{-5}$) to further likelihood ratio test procedure. We call this method MBLASSO. The flow chart is shown in Figure 1.

More specifically, MBLASSO works as follows:

- Step 1: Correct the initial phenotype vector (y) by the fixed effects, which indicate the population structure in our model.
- Step 2: Calculate the Pearson correlation of the *i*th SNP with the corrected phenotype ($y^{{}^{\prime }}$), that is,
  (10) $$\omega_{i}=\rho_{X_{i},y^{{}^{\prime }}}=\frac{\sum_{j=1}^{n}\left(x_{ji}-\bar{x_{i}}\right)\left(y_{j}^{{}^{\prime }}-\bar{y^{{}^{\prime }}}\right)}{\sqrt{\sum_{j=1}^{n}{\left(x_{ji}-\bar{x_{i}}\right)}^{2}}\cdot \sqrt{\sum_{j=1}^{n}{\left(y_{j}^{{}^{\prime }}-\bar{y^{{}^{\prime }}}\right)}^{2}}}$$
  where $x_{ji}$ is the *i*th SNP genotype value of the *j*th individual, $y_{j}^{{}^{\prime }}$ is the corrected phenotype value of the *j*th individual, $\bar{x_{i}}$ is the average of the genotype value of the *i*th SNP, $\bar{y^{{}^{\prime }}}$ is the mean of the corrected phenotype value of all individuals, and $\omega ={\left(\omega_{1},\omega_{2},\ldots ,\omega_{p}\right)}^{T}$ is a vector of Pearson correlation coefficients.
- Step 3: Sort the components of vector ω in descending order and define a subset:
  (11) $$\Omega =\{1\leq i\leq p:|\omega_{i}|\;\mathrm{is}\;\mathrm{among}\;\mathrm{the}\;\left(n-1\right)\;\mathrm{largest}\;\mathrm{of}\;\mathrm{all}\}$$
  where n−1 is one of the two sizes recommended by Fan and Lv [11], and it is more appropriate in our work. Suppose that there are $k_{1}$ SNPs corresponding to Ω, $k_{1}\geq n-1$, for the reason that more than one SNP may share a common Pearson correlation coefficient; the subset consisting of these SNPs is denoted as $A_{1}=\left\{X_{jm_{1}},X_{jm_{2}},\ldots ,X_{jm_{k_{1}}}\right\}$, $m_{1},m_{2},\ldots ,m_{k_{1}}$ are the orders of the $k_{1}$ selected SNPs in all the *p* SNPs. Then implement SCAD to estimate the effects. Select the SNPs with nonzero effect to form another subset $A_{2}=\left\{X_{jl_{1}},X_{jl_{2}},\ldots ,X_{jl_{k_{2}}}\right\}\subseteq A_{1}$, $k_{2}\leq k_{1}$, and $\left\{l_{1},l_{2},\ldots ,l_{k_{2}}\right\}\subseteq \left\{m_{1},m_{2},\ldots ,m_{k_{1}}\right\}$. The SNPs in $A_{2}$ correspond to Type I in Pearson correlation screening. This Pearson correlation based SIS followed by SCAD is called SIS-SCAD [11].
- Step 4: Undertake ISIS-SCAD [11] to revive those non-negligible SNPs that are single uncorrelated but jointly correlated with phenotype, only one iteration is implemented here. Firstly correct the phenotype in Step 1 ($y^{{}^{\prime }}$) by the $k_{2}$ SNPs selected by SIS-SCAD in Step 3, that is,
  (12) $$y^{{}^{\prime \prime }}=y^{{}^{\prime }}-\sum_{t=1}^{k_{2}}X_{l_{t}}\beta_{l_{t}}$$
  where $\beta_{l_{t}}$ is estimated by SCAD, and then repeat SIS-SCAD to the rest of the $p-k_{2}$ SNPs, which results in another subset of $k_{3}$ SNPs, $A_{3}=\left\{X_{js_{1}},X_{js_{2}},\ldots ,X_{js_{k_{3}}}\right\}$. The SNPs in $A_{3}$ correspond to Type II in Pearson correlation screening. The union of the two disjoint subsets $A_{2}$ and $A_{3}$ is denoted as A, $A=A_{2}⋃A_{3}$, whose size is *k*, $k=k_{2}+k_{3}$.
- Step 5: Under the same conditions as in Step 2, calculate the mutual information of the *i*th SNP and the corrected phenotype ($y^{{}^{\prime }}$) by
  (13) $$\psi_{i}=I\left(X_{i},y^{{}^{\prime }}\right)=\sum_{j=1}^{n}p\left(x_{ji},y_{j}^{{}^{\prime }}\right)\cdot \log\frac{p(x_{ji},y_{j}^{{}^{\prime }})}{p\left(x_{ji}\right)p\left(y_{j}^{{}^{\prime }}\right)}$$
  and $\psi ={\left(\psi_{1},\psi_{2},\ldots ,\psi_{p}\right)}^{T}$ is a vector of mutual information for all of the *p* SNPs with the corrected phenotype. $p(x_{ji},y_{j}^{{}^{\prime }})$ is the joint probability, $p(x_{ji})$ and $p(y_{j}^{{}^{\prime }})$ are the marginal probabilities of $x_{ji}$ and $y_{j}^{{}^{\prime }}$, separately.
- Step 6: Similar to Step 3, sort the components of vector ψ in descending order and define another subset:
  (14) $$\Psi =\{1\leq i\leq p:\psi_{i}\;\mathrm{is}\;\mathrm{among}\;\mathrm{the}\;\left(n-1\right)\;\mathrm{largest}\;\mathrm{of}\;\mathrm{all}\}$$
  Assume that $\tau_{1}$ SNPs corresponding to Ψ, $\tau_{1}\geq n-1$, because more than one SNP may share a public mutual information with phenotype. The subset is $B_{1}=\left\{X_{jh_{1}},X_{jh_{2}},\ldots ,X_{jh_{\tau_{1}}}\right\}$. Then use SCAD to estimate the effects of SNPs in $B_{1}$ and select the SNPs with nonzero effect to constitute a new subset $B_{2}=\left\{X_{jr_{1}},X_{jr_{2}},\ldots ,X_{jr_{\tau_{2}}}\right\}\subseteq B_{1}$, $\tau_{2}\leq \tau_{1}$, and $\left\{r_{1},r_{2},\ldots ,r_{\tau_{2}}\right\}\subseteq \left\{h_{1},h_{2},\ldots ,h_{\tau_{1}}\right\}$. The SNPs in $B_{2}$ correspond to Type I in mutual information screening. We call this mutual information based SIS followed by SCAD as MI-SIS-SCAD.
- Step 7: Refering to Step 4, correct the phenotype in Step 1 ($y^{{}^{\prime }}$) by $\tau_{2}$ SNPs selected by MI-SIS-SCAD, and repeat MI-SIS-SCAD once for to the remaining of the $p-\tau_{2}$ SNPs, which generates a subset of $\tau_{3}$ SNPs, $B_{3}=\left\{X_{jt_{1}},X_{jt_{2}},\ldots ,X_{jt_{\tau_{3}}}\right\}$. The SNPs in $B_{3}$ correspond to Type II in mutual information screening. The union of the disjoint subsets $B_{2}$ and $B_{3}$ is denoted as B, $B=B_{2}⋃B_{3}$, the size of which is τ, $\tau =\tau_{2}+\tau_{3}$. We call this process as MI-ISIS-SCAD.
- Step 8: Gather the SNPs selected from Steps 4 and 7 and remove the reduplicated ones. Then obtain a new subset of SNPs, that is, C=A⋃B, the size of which is ν=k+τ.
- Step 9: Use EM-BLASSO to estimate the effect of the ν SNPs from C and further eliminate the SNPs with zero effect, the source code for EM-BLASSO can be found at https://CRAN.R-project.org/package=mrMLM, where we can also download the program of ISIS EM-BLASSO. Note that the phenotype vector in this step refers to the original one (y).
- Step 10: Apply the likelihood ratio test to identify the true QTNs, and set the significant criterion as LOD≥3.

## 3. Results

### 3.1. The Overlap Ratio between Pearson Correlation and Mutual Information Based Screening in MBLASSO

To illustrate the necessity of considering the correlation measured in mutual information between the SNPs and phenotype, we calculated the overlap ratio and average number of SNPs selected by Pearson correlation and mutual information in the first variable selection stage. The SNPs selected by Pearson correlation and mutual information can be divided into two types (Types I and Type II), respectively. We found that each type of screening obtains phenotype-associated SNPs without large overlapping (Table 1), which suggests that the SNPs from our MBLASSO method may have more associations with phenotype than ISIS EM-BLASSO.

### 3.2. Statistical Power for QTN Detection

**Figure 2:**
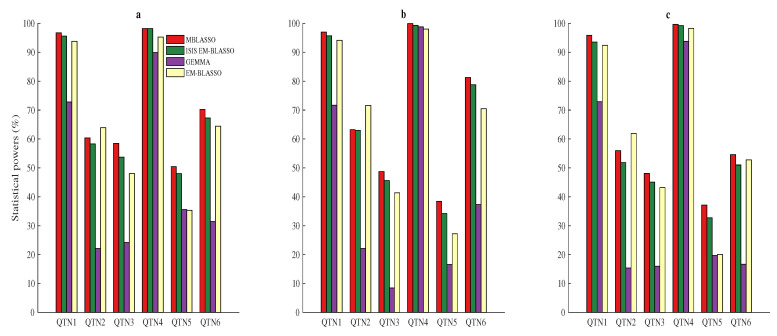
Statistical powers for the six simulated QTNs in three simulation scenarios. (**a**) only six QTNs’ additive effects; (**b**) six QTNs’ additive effects and polygenic background effect; and (**c**) six QTNs’ additive effects and three other pairs of QTNs’ epistatic effects.

The power for the *i*th QTN is: $power_{i}=\ell_{i}/1000,i=1,2,3,4,5,6$, where $\ell_{i}$ is the frequency that *i*th hypothetical QTN is successfully detected among all 1000 repetitions. A detected SNP within 1kb of the candidate QTN is regarded as true QTN [6,25,30]. In three simulations, powers of the six QTNs in MBLASSO are highest, except the second QTN powers are lower than those of EM-BLASSO (Figure 2a–c and Tables S1–S3). The average powers of MBLASSO are 72.4, 71.4, and 65.2 (%) in three simulations, respectively. They are improved by 26.4, 28.9, and 26.1 (%) compared to GEMMA; 5.6, 4.3, and 3.8 (%) compared to EM-BLASSO; and 2.2, 2.0, and 3.0 (%) compared to ISIS EM-BLASSO. We supposed four QTNs (QTN2, QTN3, QTN5, and QTN6) with the same 5% heritability, but the detection powers of QTN5 are much lower than three other values for MBLASSO, ISIS EM-BLASSO, and EM-BLASSO (Figure 2a–c and Tables S1–S3). To measure the robustness of methods, we used the standard deviation of powers across the four QTNs, which was proposed by Ren et al. [30]. In Simulation 1, the standard deviations for MBLASSO, ISIS EM-BLASSO and EM-BLASSO are 8.14, 8.16 and 13.99, respectively, indicating the best stability of MBLASSO. The stability comparisons in Simulations 2 and 3 are the same as that in Simulation 1. Therefore, MBLASSO improves the power and has best stability in different scenarios. A Violin plot of average statistical powers for MBLASSO in three simulation scenarios is shown in Figure S1a.

### 3.3. Average Accuracy for QTN Effects

**Figure 3:**
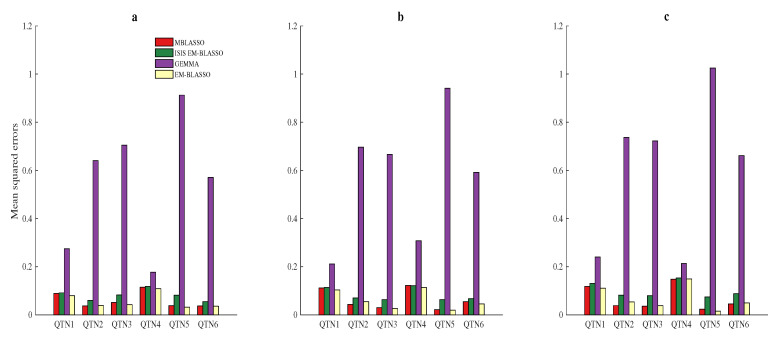
Average mean squared errors (MSEs) for the six simulated QTNs in three simulation scenarios. The description of (**a**–**c**) is the same as that in Figure 2.

Mean squared error (MSE) was used to quantify the bias of effect estimation. The MSE of the *i*th QTN is: $MSE_{i}=\frac{1}{1000}\sum_{j=1}^{1000}{\left({\hat{\beta }}_{ij}-\beta_{i}\right)}^{2}$, i=1,2,3,4,5,6, where ${\hat{\beta }}_{ij}$ is the effect of the *i*th QTN in the *j*th repetition, and $\beta_{i}$ is the theoretical effect of the *i*th QTN. The smaller the MSE is, the better the accuracy of the algorithm is. We applied the average MSE of the six QTNs to totally measure the accuracy of different algorithms. They are 0.0610, 0.0812, 0.5467 and 0.0561 for MBLASSO, ISIS EM-BLASSO, GEMMA and EM-BLASSO, respectively, in Simulation 1, the similar case is shown in Simulation 2, and the average MSE for MBLASSO is the lowest in Simulation 3 (Figure 3a–c and Tables S1–S3), indicating the better estimation accuracy of MBLASSO on the whole. A violin plot of average MSEs for MBLASSO in three simulation scenarios is shown in Figure S1b.

### 3.4. Type 1 Error Ratio

**Figure 4:**
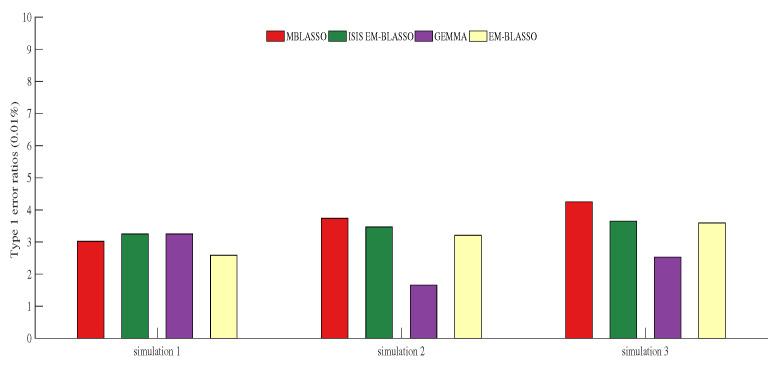
Type 1 error ratios (0.01%) in three simulation scenarios. The descriptions of Simulations 1–3 corresponding to (**a**–**c**) in Figure 2.

Type 1 error ratio, also known as false positive ratio, is an important problem that needs to be overcome in GWAS. In Simulation 1, they are 0.0302%, 0.0325%, 0.0325% and 0.0259% for MBLASSO, ISIS EM-BLASSO, GEMMA, and EM-BLASSO, respectively, and GEMMA has the lowest Type 1 error ratio in Simulations 2 and 3 (Figure 4). Note that all Type 1 error ratios are less than 0.05% (Figure 4 and Tables S1–S3), which indicates that all the four algorithms ensure the Type 1 error is at a very low level. A violin plot of Type 1 error ratios for MBLASSO in three simulation scenarios is shown in Figure S1c.

### 3.5. Computational Efficiency

The computing time of MBLASSO is longer than that of ISIS EM-BLASSO, because it needs additional computation of mutual information between all the SNPs and phenotype, but it takes less time than EM-BLASSO. For example, in Simulation 1, MBLASSO finishes the analysis of 199 individuals with 10,000 SNPs for 1000 repetitions in 4.12 h, ISIS EM-BLASSO takes 2.90 h, GEMMA spends 2.20 h, and while EM-BLASSO needs 28.86 h for the same dataset (Table S1). The specific hours spent on the other two simulations are largely identical with only minor differences to those in Simulation 1 (Tables S2 and S3), and the operations of computation are on a computer of Intel Xeon E5-2640 CPU 2.40 GHz.

### 3.6. Arabidopsis Thaliana Dataset Analysis

We analyzed four flowering-time related traits (LDV, SDV, 2W, and 4W) using by MBLASSO, ISIS EM-BLASSO, GEMMA and EM-BLASSO. Suppose that the candidate genes for the traits are in the proximity of 20 kb with the associated SNPs [6,25]; MBLASSO identifies 17, 18, 17 and 18 SNPs significant associated with each of the four traits LDV, SDV, 2W, and 4W, respectively. ISIS EM-BLASSO detects 14, 18, 19, and 16 remarkable associated SNPs; GEMMA identifies 3, 5, 1, and 2 significant SNPs; and EM-BLASSO tests 3, 0, 4, and 6 SNPs, respectively. A Venn diagram showings the overlap numbers of SNPs detected by the four algorithms in the four traits is presented in Figure S2.

To measure the model fitting degree of the detected SNPs, Akaike Information Criterion (AIC) and Bayesian Information Criterion (BIC) were computed for each trait in four various methods, where a lower value indicates a better model fitting. We can explicitly see that MBLASSO shows the lowest AIC and BIC for the four traits (Table 2), thus it is the best algorithm in model fitting, followed by ISIS EM-BLASSO, EM-BLASSO, and GEMMA.

Meanwhile, by referring to the latest GO annotation [31] for *Arabidopsis thalina* genes at www.arabidopsis.org, we extracted the known genes related to flowering-time traits and found 5, 4, 2, and 3 known genes closed to the detected SNPs with MBLASSO; 3, 2, 1, and 2 known genes with ISIS EM-BLASSO; 0, 1, 0, and 1 known genes with GEMMA; and none of known genes could be identified by using EM-BLASSO for LDV, SDV, 2W and 4W, respectively (Table 3). These results suggest that the accuracy of associations retrieved by MBLASSO are the highest.

In addition, totally 21 genes were only detected by MBLASSO, among which five genes (AT5G45830, AT5G45840, AT3G57230, AT5G15850, and AT5G04240) are in the 98 candidate genes [24], and AT5G45830 (alias: DOG1) is the gene with the highest frequency significant associated with flowering-time related phenotypes. Nearly all of the 23 flowering-time related phenotypes are associated with this gene [24]. Meanwhile, AT5G45840 (alias: MDIS1) is one of the Top 5 flowering-time related genes studied by researchers in *Arabidopsis thaliana* (www.arabidopsis.org). The detailed GWAS results are listed in Table S4.

About the computation speed, despite MBLASSO being is slower than ISIS EM-BLASSO and GEMMA, it is much faster than EM-BLASSO, for example, for the trait LDV, the time for MBLASSO is 2.31 min, ISIS EM-BLASSO requires 1.92 min, GEMMA takes 0.85 min and EM-BLASSO consumes to 183.6 min. We notice that the time costs of all the four flowering-time traits in MBLASSO, ISIS EM-BLASSO, and GEMMA are all less than 3 min (Table S5).

## 4. Discussion

MBLASSO is a GWAS method modified from ISIS EM-BLASSO, that is, iterative sure independence screening (ISIS) in the first stage of ISIS EM-BLASSO is replaced by a combination ISIS based on Pearson correlation and mutual information. We assume a subset of loci jointly affects the phenotype. In the first stage, we focus on selecting those SNPs that are likely to be highly associated. Considering some SNPs may have different correlations under various phenotypes, which are hard to measure only by Pearson correlation, so we adopt the mutual information to obtain the SNPs with potential correlation to phenotype. Meanwhile, since those SNPs individually irrelevant but jointly relevant to phenotype can be revived, this multi-objective screening process is a crucial component of our methodology to improve the statistical power. In the second stage, we apply the existing EM-BLASSO method [10], which is actually a single stage multi-locus GWAS strategy, to estimate the effects of selected SNPs and further filter out the SNPs with very small effect (<10${}^{-5}$). Finally, we use likelihood ratio test to identify the true QTNs.

In fact, the method and criterion of hypothesis testing in different approaches may be different, e.g., the Wald test is applied in RMLM [25] and original EM-BLASSO [10], the significant level is P=0.01 or 0.05, and a looser likelihood ratio test criterion LOD≥2 is employed in pLARmEB [32]. Since different significant criteria will lead to changes in results, for above three simulations, we listed the performances (average power, average MSE and Type 1 error ratio) of MBLASSO in three different significant criteria (LOD=3, LOD=2 and P=0.01) in Table S6. We can see the average power increased with the decrease of LOD value, but the Type 1 error ratio and average MSE also increased. This means that with the relaxation of significant criteria, high statistical power will be achieved, while false positives will be increased and estimation accuracy will be reduced. In addition, the performances at the significant criterion P=0.01 in Wald test are between LOD=2 and 3 in likelihood ratio test. GEMMA is a single-locus GWAS approach, and the significant threshold for each test is determined by Bonferroni correction (0.05/p, *p* is the number of SNPs). MBLASSO, ISIS EM-BLASSO, and EM-BLASSO are multi-locus approaches and do not require multiple test correction.

We conducted paired t-test (also used in [6,25,30]) for statistical power and MSE between MBLASSO and three other methods in three simulation scenarios (Table S7). We can see it has significant improvements compared with ISIS EM-BLASSO and GEMMA. For the traits SDV and 2 W in real *Arabidopsis thaliana* datasets, the numbers of significant SNPs identified by MBLASSO are not more than ISIS EM-BLASSO, but the degrees of model fitting are better (Table 2); and the number of known candidate genes adjacent to the detected SNPs is still larger (Table 3), this phenomenon indicates MBLASSO may be more effective to capture the inherent relationship between SNPs and phenotype. The traditional EM-BLASSO [10] and GEMMA perform well in terms of Type 1 error ratio in the three simulations, but their performances in *Arabidopsis thaliana* dataset are worse than expected, not only achieving the worse model fitting performance but also fewer of genes are detected. On the whole, our algorithm MBLASSO is slightly slower than ISIS EM-BLASSO and GEMMA, but it is more effective and accurate for both simulation and real datasets.

## 5. Conclusions

Our algorithm MBLASSO is a modified version of ISIS EM-BLASSO; it integrates Pearson correlation and mutual information to the feature screening stage, and it considers different types of correlation between the SNPs and phenotype. In three different simulation scenarios, MBLASSO improves the statistical power and retains the higher effect estimation accuracy when comparing with three other methods. Meanwhile, the GWAS results in four flowering-time related traits are superior in model fitting; the accuracy of detected associations are the highest; and 21 genes can only be detected by MBLASSO.

## Figures and Tables

**Table 1:** Screening results based on Pearson correlation and mutual information in MBLASSO under three simulation scenarios (each cell includes the overlap ratio and average number of SNPs after screening in the parentheses).

Simulations	Pearson Correlation Screening			Mutual Information Screening		
	Type I	Type II	Total	Type I	Type II	Total
1	0.470 (15.8)	0.086 (50.4)	0.184 (66.2)	0.417 (18.2)	0.298 (15.5)	0.356 (33.7)
2	0.452 (16.6)	0.091 (50.3)	0.181 (66.9)	0.398 (19.0)	0.285 (17.5)	0.334 (36.5)
3	0.457 (14.6)	0.090 (50.8)	0.173 (65.4)	0.383 (18.4)	0.278 (17.4)	0.323 (35.8)

**Table 2:** Degree of model fitting (AIC, BIC) for SNPs identified in four flowering-time related traits for *Arabidopsis thaliana.*

Traits	MBLASSO		ISIS EM-BLASSO		GEMMA		EM-BLASSO	
	AIC	BIC	AIC	BIC	AIC	BIC	AIC	BIC
LDV	−360.543	−307.436	−318.966	−275.230	1312.693	1322.065	−113.638	−104.266
SDV	−169.269	−114.028	−140.485	−85.245	1356.907	1372.251	149.095	149.095
2W	−103.363	−51.957	−65.172	−7.718	584.000	587.024	148.247	160.342
4W	−124.109	−74.084	−98.993	−54.527	1253.281	1258.839	22.893	39.568

**Table 3:** The accuracy of detected associations in four flowering-time related traits for *Arabidopsis thaliana* (the number behind slash in each cell is the count of detected SNPs, and the number in front of slash is the count of known genes in GO annotation),

Traits	MBLASSO	ISIS EM-BLASSO	GEMMA	EM-BLASSO
LDV	5/17	3/14	0/3	0/3
SDV	4/18	2/18	1/5	0/0
2W	2/17	1/19	0/1	0/4
4W	3/18	2/16	1/2	0/6

## Supplementary Materials

The following are available online at https://www.mdpi.com/1099-4300/22/3/329/s1.
