# Supplementary material for: A Two-Stage Mutual Information Based Bayesian Lasso Algorithm for Multi-Locus Genome-Wide Association Studies
Source: Entropy (Basel). 2020 Mar 13;22(3):329. doi: 10.3390/e22030329 (PMC7516787; doi:10.3390/e22030329)
Supplement: Supplementary file 1 [file entropy-22-00329-s001.pdf]

A two-stage mutual information based Bayesian Lasso algorithm for multi-locus genome-wide association studies

Hong-Ping Guo[1,2], Zu-Guo Yu[1,3,*], Ji-Yuan An[4], Guo-Sheng Han[1], Yuan-Lin Ma[1], and Run-Bin Tang[1]

[1] Key Laboratory of Intelligent Computing and Information Processing of Ministry of Education and Hunan Key Laboratory for Computation and Simulation in Science and Engineering, Xiangtan University, Hunan 411105, China.

[2] School of Mathematics and Computer Science, Hanjiang Normal University, Hubei 442000, China.

[3] School of Electrical Engineering and Computer Science, Queensland University of Technology, QLD 4001, Australia.

[4] Centre for Tropical Crops and Biocommodities, Queensland University of Technology, Brisbane, QLD 4001, Australia

[*]Corresponding author: yuzuguo@aliyun.com

Table S1: The results of the first simulation scenario (with only six additive QTNs) by MBLASSO, ISIS EM-BLASSO, GEMMA and EM-BLASSO

QTN	True values				MBLASSO			ISIS EM-BLASSO			GEMMA			EM-BLASSO		
	Position (bp)	Chr	r^2	Effect	Effect	MSE	Power	Effect	MSE	Power	Effect	MSE	Power	Effect	MSE	Power
1	11298364	1	0.1	1.476	1.4033	0.0889	0.967	1.4044	0.0913	0.956	1.9474	0.2748	0.728	1.4087	0.0797	0.938
2	11655607	1	0.05	1.044	1.0772	0.0369	0.604	1.0847	0.0598	0.583	1.8344	0.6408	0.221	1.0559	0.0388	0.639
3	5066968	2	0.05	1.044	1.1432	0.0511	0.585	1.1419	0.0821	0.537	1.8674	0.7045	0.242	1.1555	0.0424	0.480
4	5134228	2	0.15	1.808	1.6847	0.1143	0.982	1.6819	0.1177	0.982	2.1589	0.1766	0.899	1.6796	0.1085	0.953
5	5464675	2	0.05	1.044	1.1718	0.0383	0.504	1.1705	0.0815	0.480	1.9861	0.9127	0.356	1.1731	0.0314	0.353
6	6137189	2	0.05	1.044	1.0467	0.0364	0.702	1.0484	0.0546	0.673	1.7837	0.5705	0.314	1.0284	0.0358	0.644
**Time Taken (Hrs) (199 individuals with 10000 SNPs 1000 replicates)**					4.12			2.90			2.20			28.86		
**Empirical Type 1 Error (0.01%)**					3.02			3.25			3.25			2.59		

Chr: chromosome, r^2: the proportion of phenotypic variance explained by each QTL, MSE: mean square error.

Table S2: The results of the second simulation scenario (with six additive QTNs and polygenic background explaining 0.092 of the phenotypic variance) by MBLASSO, ISIS EM-BLASSO, GEMMA and EM-BLASSO

QTN	True values				MBLASSO			ISIS EM-BLASSO			GEMMA			EM-BLASSO		
	Position (bp)	Chr	r^2	Effect	Effect	MSE	Power	Effect	MSE	Power	Effect	MSE	Power	Effect	MSE	Power
1	11298364	1	0.1	1.617	1.4462	0.1112	0.970	1.4533	0.1137	0.957	2.0249	0.2108	0.717	1.4504	0.1029	0.941
2	11655607	1	0.05	1.143	1.0779	0.0435	0.632	1.0804	0.0699	0.630	1.9674	0.6968	0.221	1.0672	0.0543	0.716
3	5066968	2	0.05	1.143	1.1494	0.0292	0.487	1.1538	0.0624	0.456	1.9481	0.6659	0.085	1.1660	0.0262	0.414
4	5134228	2	0.15	1.981	2.0037	0.1218	0.999	1.9908	0.1206	0.993	2.4673	0.3080	0.988	1.9780	0.1135	0.980
5	5464675	2	0.05	1.143	1.1234	0.0211	0.384	1.1371	0.0631	0.342	2.1039	0.9415	0.166	1.1361	0.0189	0.272
6	6137189	2	0.05	1.143	1.1654	0.0547	0.813	1.1664	0.0665	0.788	1.8970	0.5917	0.373	1.1734	0.0449	0.705
**Time Taken (Hrs) (199 individuals with 10000 SNPs 1000 replicates)**					4.25			2.97			2.31			28.52		
**Empirical Type 1 Error (0.01%)**					3.74			3.47			1.66			3.21		

Chr: chromosome, r^2: the proportion of phenotypic variance explained by each QTL, MSE: mean square error.

Table S3: The results of the third simulation scenario (with six additive QTNs and three other pairs of epistatic QTNs each explaining 0.05 of the phenotypic variance) by MBLASSO, ISIS EM-BLASSO, GEMMA and EM-BLASSO

QTN	True values				MBLASSO			ISIS EM-BLASSO			GEMMA			EM-BLASSO		
	Position (bp)	Chr	r^2	Effect	Effect	MSE	Power	Effect	MSE	Power	Effect	MSE	Power	Effect	MSE	Power
1	11298364	1	0.1	1.731	1.6031	0.1182	0.959	1.5990	0.1298	0.935	2.1624	0.2406	0.729	1.5904	0.1103	0.924
2	11655607	1	0.05	1.224	1.1301	0.0384	0.560	1.1238	0.0813	0.518	2.0730	0.7373	0.154	1.0940	0.0531	0.619
3	5066968	2	0.05	1.224	1.3090	0.0363	0.481	1.2958	0.0789	0.451	2.0657	0.7219	0.160	1.3046	0.0379	0.432
4	5134228	2	0.15	2.120	1.9692	0.1482	0.997	1.9629	0.1526	0.992	2.5026	0.2132	0.938	1.9702	0.1488	0.983
5	5464675	2	0.05	1.224	1.2765	0.0232	0.371	1.2847	0.0740	0.327	2.2239	1.0249	0.197	1.2891	0.0155	0.200
6	6137189	2	0.05	1.224	1.0823	0.0456	0.546	1.0849	0.0872	0.510	2.0237	0.6614	0.167	1.0602	0.0498	0.528
**Time Taken (Hrs) (199 individuals with 10000 SNPs 1000 replicates)**					4.16			3.04			2.27			29.48		
**Empirical Type 1 Error (0.01%)**					4.25			3.65			2.53			3.59		

Chr: chromosome, r^2: the proportion of phenotypic variance explained by each QTL, MSE: mean square error.

Table S4: The GWAS results of four flowering-time related traits in *Arabidopsis thaliana* by MBLASSO, ISIS EM-BLASSO, GEMMA and EM-BLASSO

Trait	Gene Name	Gene ID	Chr	SNP Position	MBLASSO			ISIS EM-BLASSO			GEMMA			EM-BLASSO			Distance to Gene (bp)
					Effect	LOD	r2	Effect	LOD	r2	Effect	P-value	r2	Effect	LOD	r2	
**LDV**	BRX	AT1G31880	1	11469582	0.040	10.94	2.20										19393
	CYP76C6	AT1G33720	1	12204210	0.046	5.74	1.21										-16687
	**VIP5**	**AT1G61040**	1	22495012	0.021	3.76	0.61	0.028	4.34	1.10							9043
	GLI1	AT1G80460	1	30244136	-0.025	5.57	0.94	-0.018	3.04	0.47							-2609
	ADR1	AT1G33560	1	12178184				-0.035	5.03	1.51							6179
	BCA4	AT1G70410	1	26552074										0.057	5.27	5.08	13741
	HEN2	AT2G06990	2	2886573	0.029	4.79	1.28	0.042	7.58	2.63							-8367
	CER8	AT2G47240	2	19396129	-0.023	3.74	0.72										0
	AT2G13960	AT2G13960	2	5851520				0.022	3.45	0.75							-8065
	**CKB4**	**AT2G44680**	2	18446546							0.157	8.98e-8	17.88				18189
	PDLZ2	AT3G05630	3	1625772	0.040	9.65	2.49	0.036	5.82	1.95							-9428
	AIF2	AT3G06590	3	2069660	0.026	3.16	0.93										14246
	BBC1	AT3G49010	3	18153207	-0.050	8.27	2.49										-13562
	**AGL16**	**AT3G57230**	3	21181219	-0.024	3.81	0.80										287

	**INT6**	**AT3G5729**0	3	21181219	-0.024	3.81	0.80										-15331
	PIP1A	AT3G61430	3	22743451	-0.031	7.16	1.36										8116
	ARA6	AT3G54840	3	20317067				0.032	4.22	1.21							-1345
	FBA8	AT3G52930	3	19639916										0.060	5.88	5.63	10843
	ORP1C	AT4G08180	4	5188778	-0.029	5.23	1.04										15116
	CYP707A1	AT4G19230	4	10528698	-0.037	8.54	1.68	-0.045	9.22	2.48							4737
	IAA29	AT4G32280	4	15574901				0.030	4.47	1.07							-8486
	PUX4	AT4G04210	4	2043173							-0.163	6.22e-9	11.98				11320
	**MDIS1**	**AT5G15840**	5	5186340	-0.025	4.45	1.02										13582
	**COL2**	**AT5G15850**	5	5186340	-0.025	4.45	1.02										8443
	PSY	AT5G17230	5	5682001	0.028	5.37	0.90										19136
	DOG1	AT5G45830	5	18582703	-0.048	7.97	2.06										-6779
	LOB	AT5G63090	5	25290046	-0.059	9.16	2.33	-0.038	3.46	0.94	0.126	1.53e-9	10.51				-18526
	AT5G21090	AT5G21090	5	7170292				-0.033	5.47	1.48							3037
	**ATPI4K*3**	**AT5G24240**	5	8248050				0.035	3.25	0.93							13900
	DOG1	AT5G45830	5	18599929				-0.045	6.67	2.90							8529
	**NF-YC4**	**AT5G63470**	5	25407568				-0.057	6.33	2.82							-8133
	IMS2	AT5G23020	5	7704503										-0.071	6.05	8.09	-13618

**SDV**	**AGL104**	**AT1G22130**	1	7792894	-0.091	6.66	2.72	-0.117	11.89	4.50							-19493
	ATNCED5	AT1G30100	1	10579398	0.071	6.79	2.39	0.065	6.65	1.99							6033
	JAZ8	AT1G30135	1	10611240	-0.045	3.55	0.84	-0.056	6.22	1.31							14001
	**AGL50**	**AT1G59810**	1	22003369	-0.053	5.34	1.59										-5235
	AtbHLH112	AT1G61660	1	22734208	0.069	9.18	2.56	0.041	4.87	0.89							-19507
	NAP3	AT1G67940	1	25460666	-0.046	3.91	1.11	-0.057	6.20	1.71							-17004
	**FLM**	**AT1G77080**	1	28965510	-0.078	9.99	3.59	-0.077	11.60	3.48							5414
	INO	AT1G23420	1	8309507				-0.046	3.92	0.78							-7889
	GA2OX7	AT1G50960	1	18887008							0.262	2.46e-9	13.7				-2541
	HPGT3	AT2G25300	2	10765594	-0.062	7.50	2.11	-0.061	8.72	2.06							-6100
	ATKA02	AT2G32440	2	13774332	-0.049	4.80	1.38										-256
	AHG3	AT3G11410	3	3564586	0.069	8.55	2.75	0.042	4.23	1.03							-19297
	AT3G11930	AT3G11930	3	3777956	-0.059	4.35	1.05	-0.056	4.49	0.95							262
	AT3G42570	AT3G42570	3	14689623	-0.031	3.13	0.52	-0.050	6.14	1.38							0
	ARA6	AT3G54840	3	20315875	0.077	5.73	1.71										-2537
	DMS3	AT3G49250	3	18245441				-0.090	5.48	1.73							-12820
	EPFL9	AT4G12970	4	7586463	-0.095	6.12	2.12										0
	ADA2B	AT4G16420	4	9282816	-0.033	3.69	0.62	-0.031	3.57	0.53							16730

	DLO1	AT4G10500	4	6471196				-0.040	3.74	0.57							-19821
	AT4G20460	AT4G20460	4	11038635				0.028	3.52	0.45							5958
	SAUR9	AT4G36110	4	17103594				0.068	6.01	1.35							12769
	**ELF6**	**AT5G04240**	5	1164843	-0.064	4.83	1.63										-4701
	YAK1	AT5G35980	5	14131434	0.095	9.83	4.38	0.121	18.26	7.07							0
	STPL	AT5G45980	5	18654302	0.050	4.61	1.44	0.043	4.52	1.10	0.170	2.94e-10	16.82				3466
	TUA3	AT5G19770	5	6685573							0.318	2.84e-8	30.33				994
	HB-7	AT5G46880	5	19044037							0.206	2.37e-8	9.46				8306
	**ICU2**	**AT5G67100**	5	26794176							0.147	1.24e-7	11.00				9072
**2W**	KNAT6	AT1G23380	1	8292995	-0.093	9.03	1.83	-0.090	9.35	1.70							-4296
	BCA3	AT1G23730	1	8392979	0.099	7.13	1.24	0.073	5.12	0.69							-2578
	**CRY2**	**AT1G04400**	1	1201395	0.063	3.93	0.67										12878
	LEC1	AT1G21970	1	7749248				-0.142	10.45	2.10							19631
	DD46	AT1G22015	1	7752174				-0.067	3.65	0.61							0
	SIG1	AT1G64860	1	24111298				-0.038	3.22	0.35							10346
	CYP707A2	AT2G09090	2	12481808	-0.074	4.52	0.61	-0.049	3.13	0.26							-13043
	**SPA1**	**AT2G46340**	2	19008023	-0.078	5.20	1.12	-0.064	4.35	0.75							-14150
	PHT5	AT2G32830	2	13912889							0.342	2.45e-8	15.34				-14810

	JAL35	AT3G16470	3	5579959	-0.168	11.37	2.96	-0.172	12.76	3.12							-15970
	HYH	AT3G17609	3	6006221	0.064	5.87	0.97	0.066	6.43	1.03							-17645
	MDR1	AT3G08860	3	10855475	0.095	6.05	1.60	0.089	5.72	1.40							14563
	SPP1	AT3G58490	3	21613189	-0.082	5.03	0.86	-0.060	3.42	0.46							-19623
	ORG3	AT3G56980	3	21079518										-0.258	6.29	12.93	-7028
	ETC3	AT4G01060	4	454954	-0.075	6.86	1.34	-0.073	7.31	1.27							-5518
	SULTR;2	AT4G02700	4	1196903	-0.076	6.19	1.23	-0.087	8.50	1.62							3292
	GBF1	AT4G36730	4	17324466	0.068	5.86	1.08	0.057	4.95	0.77				0.090	4.29	1.89	11978
	GAE5	AT4G12250	4	7277805										-0.091	4.61	1.97	-11387
	ERF106	AT5G07580	5	2407884	0.075	5.35	0.95	0.077	6.59	0.99							7283
	KAN	AT5G16560	5	5423605	-0.091	9.69	1.97	-0.081	8.39	1.54				-0.129	6.16	3.95	12181
	FASS	AT5G18580	5	6189332	-0.083	5.94	1.31	-0.080	6.19	1.21							10931
	SPL7	AT5G18830	5	6289819	-0.070	5.20	1.11	-0.049	3.61	0.55							9138
	MIR319	AT5G41663	5	16642641	0.095	7.34	1.29	0.084	7.04	1.02							-17828
**4W**	ELP	AT1G05850	1	1776468	-0.123	7.85	1.32	-0.148	9.65	1.89							7773
	MOM	AT1G08060	1	2492674	-0.059	6.75	0.86	-0.059	5.59	0.86							-9068
	PIN7	AT1G23080	1	8182820	-0.104	12.64	1.75	-0.099	10.62	1.58				-0.092	5.62	1.38	0
	APC6	AT1G78770	1	29607950	0.106	6.41	1.39	0.139	9.60	2.38							-8969

	CYP79F2	AT1G16400	1	5587466				-0.043	3.54	0.40							-17693
	CPK33	AT1G50700	1	18785200				-0.048	4.11	0.52							618
	GLX1	AT1G11840	1	3978063							0.356	2.60e-7	23.57				-17106
	AT2G07180	AT2G07180	2	2979725	-0.068	8.16	1.06	-0.067	7.12	1.03							-1174
	**ACG1**	**AT2G19520**	2	8455196	0.035	4.08	0.32										-740
	CYP707A2	AT2G29090	2	12481484	-0.077	5.98	0.89										-13367
	**SPA1**	**AT2G46340**	2	19009054	-0.041	3.29	0.27	-0.060	4.32	0.57							-13119
	PSBW	AT2G30570	2	13039164										-0.127	7.50	4.51	18970
	**AGL22**	**AT2G22540**	2	9588685							0.365	2.31e-8	30.00				4792
	JAL35	AT3G16470	3	5579959	-0.086	4.53	1.02							-0.201	8.97	5.55	-15970
	MDR1	AT3G28860	3	10855475	0.113	14.68	2.84	0.119	14.77	3.12							-14563
	ATVTI13	AT3G29100	3	11084600	-0.050	3.08	0.27										7060
	AT3G44050	AT3G44050	3	15833892	-0.028	3.84	0.22	-0.024	3.12	0.17							9031
	FBA8	AT3G52930	3	19639423										0.085	4.34	2.02	10350
	ORP1C	AT4G08180	4	5188778	-0.088	8.99	1.53	-0.071	5.37	1.00							15116
	CASPL1D1	AT4G15610	4	8929271	0.042	3.78	0.30										18363
	**UBP16**	**AT4G24560**	4	12687669	-0.057	5.64	0.55	-0.058	5.43	0.57							2730
	GIN2	AT4G29130	4	14332687				0.044	4.39	0.55							-19350

	ETC3	AT4G01060	4	475865										-0.160	8.50	5.42	14780
	ATPD	AT4G09650	4	6107363										0.152	6.65	5.61	5655
	KAN	AT5G16560	5	5426861	-0.106	7.86	1.23	-0.100	6.35	1.09							15437
	IRX3	AT5G17420	5	5743728	-0.116	12.95	2.27	-0.109	10.86	1.99							2263
	DOG1	AT5G45830	5	18582703	-0.075	5.83	0.77	-0.091	7.47	1.13							-6779

LDV: days to flowering under long days with vernalization; SDV: days to flowering under short days with vernalization; 2W: days to flowering under long days for 2 weeks vernalization; 4W: days to flowering under long days for 4 weeks vernalization. The bold gene names and IDs are the true genes associated with the flowering time of *Arabidopsis thaliana* in GO annotations.

Table S5: The running time (minutes) of four flowering-time related traits in *Arabidopsis thaliana* by MBLASSO, ISIS EM-BLASSO, GEMMA and EM-BLASSO on a computer with an Intel Xeon E5-2640 CPU 2.40GHz.

Trait	Sample size	MBLASSO	ISIS EM-BLASSO	GEMMA	EM-BLASSO
LDV	168	2.31	1.92	0.85	183.6
SDV	159	2.20	2.01	0.87	171.2
2W	152	2.35	2.12	0.45	175.8
4W	119	1.86	2.01	0.48	148.6

LDV: days to flowering under long days with vernalization; SDV: days to flowering under short days with vernalization; 2W: days to flowering under long days for 2 weeks vernalization; 4W: days to flowering under long days for 4 weeks vernalization. The sample size refers to the number of sample without the missing phenotype value.

Table S6: The performances (average power, average MSE, type 1 error ratio) of MBLASSO under three different significant criteria in three simulation scenarios.

Simulation	Average power			Average MSE			Type 1 error ratio (0.01%)		
	LOD=3	LOD=2	P=0.01	LOD=3	LOD=2	P=0.01	LOD=3	LOD=2	P=0.01
1	0.724	0.755	0.737	0.0610	0.0732	0.0669	3.02	4.46	3.00
2	0.715	0.740	0.725	0.0636	0.0769	0.0706	3.74	5.35	3.77
3	0.652	0.675	0.653	0.0683	0.0874	0.0765	4.25	5.94	4.37

LOD=3 and LOD=2 are two significant criteria used in likelihood ratio test, and P=0.01 is a significant criterion used in Wald test. MSE: mean square error.

Table S7: Paired t-test and their P-values for statistical power and MSE between MBLASSO and ISIS EM-BLASSO / GEMMA / EM-BLASSO in three simulation scenarios.

Cases			Simulation 1	Simulation 2	Simulation 3
Power	MBLASSO & ISIS EM-BLASSO	t-value	3.323	3.138	5.131
		P-value	0.0209*	0.0257*	0.0037**
	MBLASSO & GEMMA	t-value	5.031	4.326	4.832
		P-value	0.0040 **	0.0075**	0.0048**
	MBLASSO & EM-BLASSO	t-value	2.114	1.433	1.239
		P-value	0.0881	0.2112	0.270
MSE	MBLASSO & ISIS EM-BLASSO	t-value	-3.119	-2.688	-4.111
		P-value	0.0263*	0.0434*	0.0093**
	MBLASSO & GEMMA	t-value	-3.902	-4.001	-3.571
		P-value	0.0114*	0.0103*	0.0160*
	MBLASSO & EM-BLASSO	t-value	2.642	1.111	-0.267
		P-value	0.0459*	0.3172	0.800

* and **: significances at the 0.05 and 0.01 levels, respectively. MSE: mean square error.

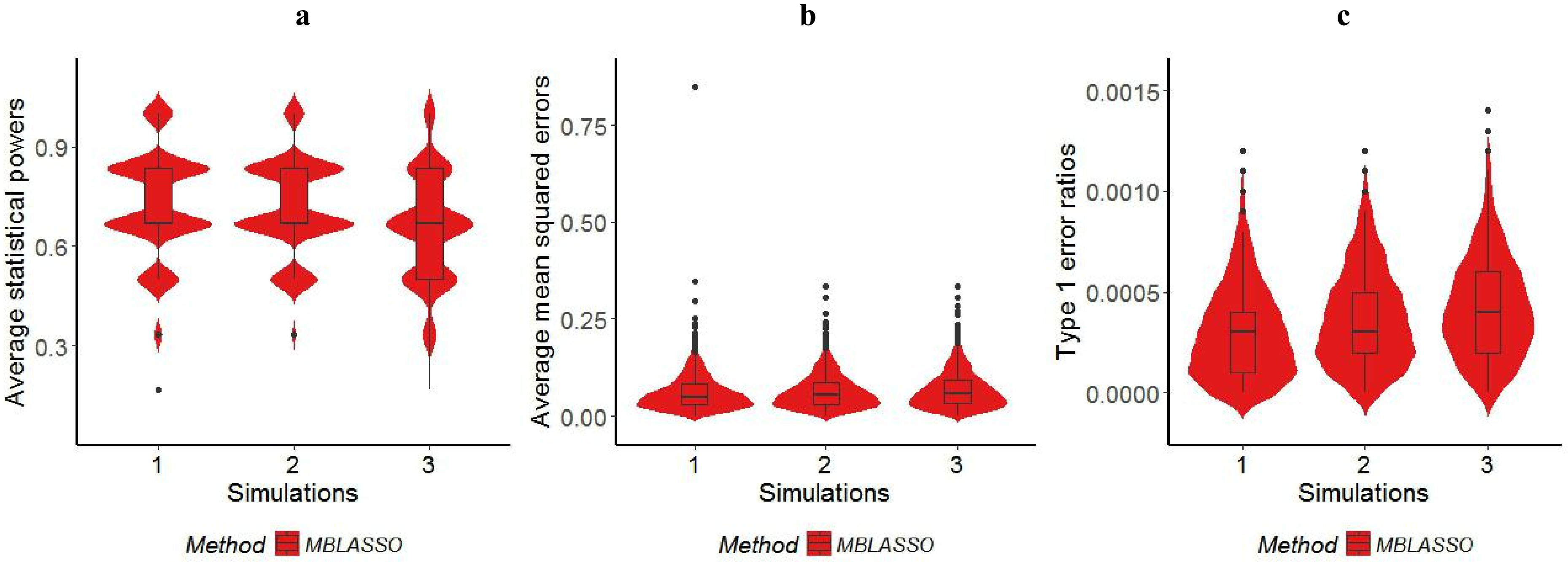

Figure S1: (a) Violin plot of average statistical powers for MBLASSO in three simulation scenarios. (b) Violin plot of average mean squared errors for MBLASSO in three simulation scenarios. (c) Violin plot of type 1 error ratios for MBLASSO in three simulation scenarios.

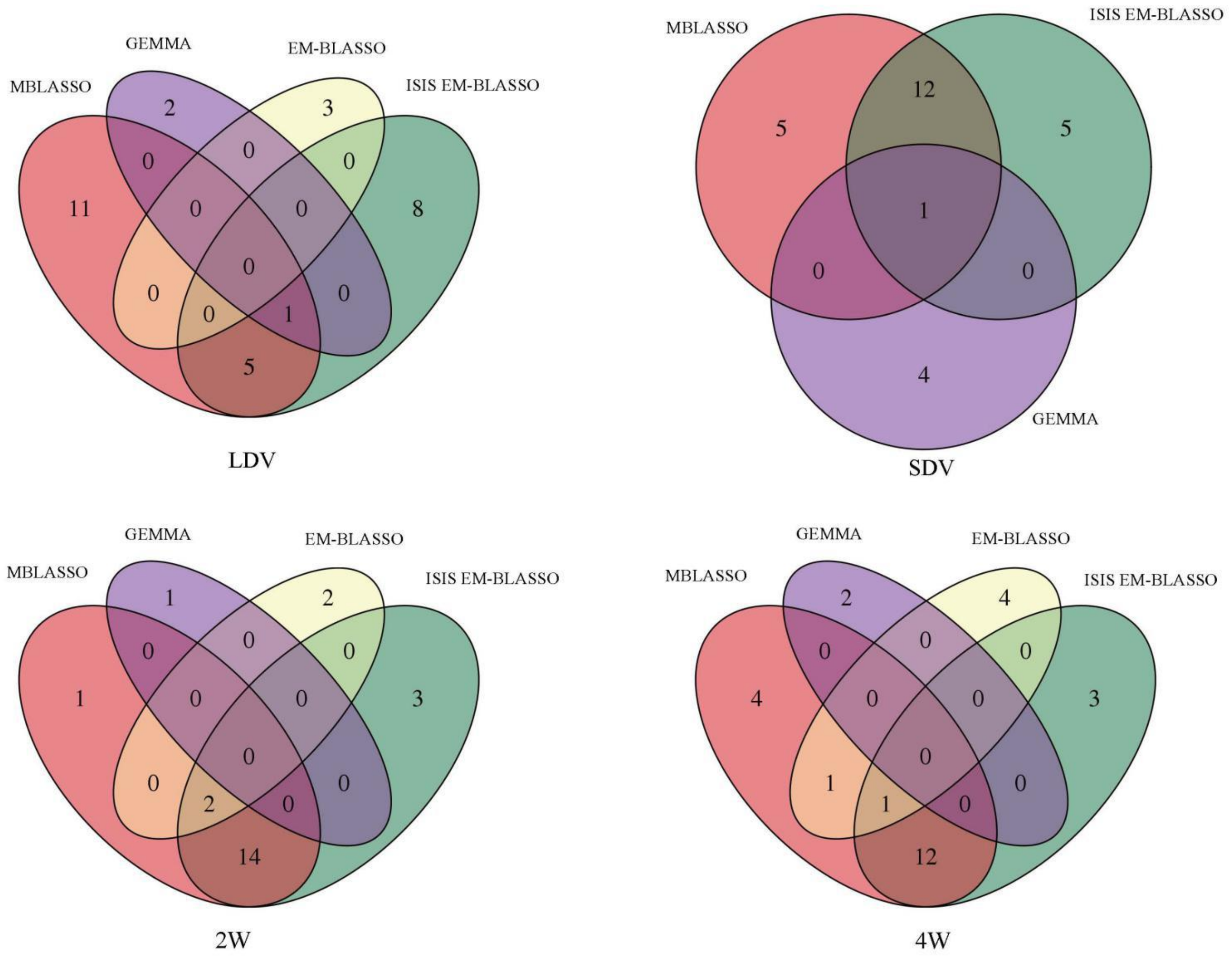

Figure S2: Venn diagram of the overlap numbers of SNPs detected by four GWAS methods in four flowering-time related traits (LDV, SDV, 2W, 4W) for *Arabidopsis thaliana*.
